# A cross-sectional study about the relationship between physical activity and sarcopenia in Taiwanese older adults

**DOI:** 10.1038/s41598-021-90869-1

**Published:** 2021-06-01

**Authors:** Yun-Chen Ko, Wei-Chu Chie, Tai-Yin Wu, Chin-Yu Ho, Wen-Ruey Yu

**Affiliations:** 1 Institute of Epidemiology and Preventive Medicine, College of Public Health, National Taiwan University, Taipei, Taiwan; 2 Department of Family Medicine, Taipei City Hospital, Yangming Branch , Taipei, Taiwan; 3 National Taiwan University Hospital, Taipei, Taiwan; 4 Taipei City Hospital, Zhongxing Branch , Taipei, Taiwan; 5 Faculty of Medicine, National Yang-Ming University, Taipei, Taiwan; 6 Department of Psychology, Soochow University, Taipei, Taiwan; 7 University of Taipei, Taipei, Taiwan

**Keywords:** Health care, Medical research, Risk factors

## Abstract

To the best of our knowledge, none of Taiwanese studies on the relationship between physical activity (PA) and sarcopenia by the latest 2019 Asian Working Group for Sarcopenia (AWGS) cutoff points of sarcopenia has been published. We used the Taiwan version of international physical activity questionnaire-short version and the 2019 AWGS diagnostic criteria of sarcopenia to examine the relationship between PA and sarcopenia in older adults. Volunteers in this cross-sectional study were recruited from those attending senior health checkup program held at a regional hospital in Taipei City from May 2019 to Sep 2019. Muscle strength was assessed by grip strength, physical performance was assessed by usual gait speed on a 6-m course, and muscle mass was measured by bioelectrical impedance analysis. Multiple logistic regression was used to analyze the relationship between PA and sarcopenia. Odds ratios and corresponding 95% confidence intervals were calculated. 565 participants were recruited and data from 500 participants were used. The study participants had a mean age of 73.87 years old, with 47% men and 53% women. 138 (27.6%) participants were classified as having sarcopenia, among which 48 (45.3%) in low PA participants and 90 (22.8%) in moderate to high PA participants. Compared with those with low PA, moderate to high PA protected against the risk of sarcopenia with the odds ratio (OR) 0.46 (95% CI 0.27–0.79, p-value = 0.005). A significant protective effect of PA on sarcopenia was found among the older adults after adjusting for sex, institutionalization, age, BMI, albumin, hemoglobin, HDL-C levels, history of cardiovascular disease, education level and alcohol drinking.

## Introduction

Sarcopenia describes an important change in body composition and function, which is characterized by age-related lean muscle mass decline and low muscle strength and/or performance. The consequences of sarcopenia are falls, fracture, disability, hospital admission or need for long-term care placement, poor quality of life, and even mortality, which lead to a heavy burden on an aging society^1–3^. Consequently, sarcopenia has been formally recognized as a muscle disease since 2016. In some countries, International Classification of Diseases, 10th Revision, Clinical Modification (ICD-10-CM) diagnosis code for sarcopenia, M62.84, could be used to bill for care^4^.

The prevalence of sarcopenia varied from 9.9 to 40.4%, depending on the definition used^5^. The definition and diagnosis of sarcopenia was inconsistent due to multiform measurements in muscle quality and quantity. In 2014, the Asian Working Group for Sarcopenia (AWGS) recommended using low muscle mass plus low muscle strength and/or low physical performance to diagnose sarcopenia with the following cut-off values: height-adjusted appendicular skeletal muscle mass as < 7.0 kg/m^2^ in men and as < 5.7 kg/m^2^ in women by using bio-electrical impedance analysis (BIA), handgrip strength as < 26 kg in men and as < 18 kg in women, and usual gait speed as < 0.8 m/s^6^. Recently, AWGS announced the 2019 diagnostic criteria of sarcopenia and revised the cut-off points of handgrip strength (< 28 kg for men) and usual gait speed (< 1.0 m/s) based on more Asian data and studies^7^.

The risk factors of sarcopenia are multifactorial, such as aging, disease, malnutrition, and inactivity, etc. Physical activity (PA) is undoubtedly a protective factor for sarcopenia. The beneficial effects of PA on sarcopenia include: reduced apoptosis, reduced oxidative stress, anti-inflammation, improved insulin-glucose dynamics, enhanced quality and quantity of muscle proteins and mitochondria, skeletal muscle hypertrophy, positive neuromuscular adaptations, and enhanced muscle blood supply^8^. In a systemic review including 37 randomized controlled studies (RCTs), PA increases muscle mass and function while interactive effect of nutrition on muscle function appears limited^9^. PA is one of the most important keys to prevent sarcopenia, which is a modifiable predictor and can improve the muscle quality and quantity^10^.

According to the World Health Organization (WHO), the definition of PA is any bodily movement produced by skeletal muscles that requires energy consumption^11^. Physical inactivity has been recognized as the fourth leading risk factor for global mortality (6% of deaths globally)^12^. WHO recommends the amount of PA should be at least 150 minutes of moderate aerobic PA or 75 minutes of vigorous aerobic one per week for older adults^13^.

PA assessment tools include report-based, monitor-based and criterion measures^14^. The majority of scientific evidence on the health benefits of PA has been accumulated with report-based measures predominantly. In many types of epidemiology studies, the main purpose was simply to classify individuals into general levels of PA participation. Report-based measures have been proven to provide sufficient accuracy to categorize individuals based on their level of PA^15^.

Among self-report questionnaires for PA measurement, international physical activity questionnaire-short version (IPAQ-S) was designed to be easily adapted in many languages and countries^16^. The validity and reliability of the Taiwan version of IPAQ-S has been verified^17,18^. Some studies have explored the relationship between PA and sarcopenia in older adults^19^. The potential confounders are aging, body mass index (BMI), gender, education level, albumin level, insulin resistance, lipid profiles, hemoglobin, uric acid, alcohol drinking, smoking, and institutionalization^10,19^.

The definition of sarcopenia and the ways to evaluate PA were inconsistent. Moreover, there were still discrepancies between some associated factors and sarcopenia. To the best of our knowledge, none of studies on the relationship between PA and sarcopenia by the latest AWGS cutoff points of sarcopenia has been published, even though in the Asian populations. Therefore, the primary aim of this study is to use the Taiwan version of IPAQ-S and the 2019 AWGS diagnostic criteria of sarcopenia to examine the relationship between PA and the presence of sarcopenia in older adults. The secondary aim is to identify other associated factors of sarcopenia. We would test the hypothesis that higher physical activity would associated with lower prevalence of sarcopenia.

## Methods

### Study population

We conducted a cross-sectional study. 565 volunteers were recruited from those attending the senior health checkup program held at a regional hospital in Taipei City from May 2019 to Sep 2019. The inclusion criteria were: 1. Age ≥ 65 years old. 2. Could perform the physical evaluation and complete the IPAQ-S. Those who could not perform the physical evaluation or answer the IPAQ-S were excluded. In addition, we also excluded participants with invalid or missing data while we did data analysis. The study had been approved by the Taipei City Hospital Research Ethics Committee with the case number TCHIRB-10801017 and all the participants provided written informed consents. All methods were carried out in accordance with relevant guidelines and regulations of the Taipei City Hospital Research Ethics Committee.

### Measurements

#### Assessment of sarcopenia

We defined sarcopenia according to the 2019 AWGS diagnostic criteria^7^. Muscle strength was assessed by grip strength, which was measured by a dynamometer (BASELINE, model 12-0286), and low grip strength was defined as < 28 kg in men and < 18 kg in women. Physical performance was assessed by usual gait speed (m/s) on a 6-m course, and a slow walking speed was defined as slower than 1.0 m/s. Muscle mass was measured by BIA (InBody270). The height-adjusted ASM (ASMI) was defined as appendicular skeletal muscle mass (ASM) divided by height squared. Low muscle mass was defined as ASMI < 7.0 kg/m^2^ in men and < 5.7 kg/m^2^ in women^7^. The reliability and validity of the tools had been appraised in a systematic review^20^. In a Chinese study involving 944 community-dwelling adults aged ≥ 60 years^21^, quite high correlation coefficient between the BIA- (InBody720) and DXA-measured ASMs revealed that the tool was suitable for body composition monitoring.

#### Assessment of physical activity

PA was assessed using the Taiwan version of IPAQ-S. We have already obtained permission to use the questionnaire (The information about applying for the permission and the Taiwan version of IPAQ-S: https://www.hpa.gov.tw/Pages/Detail.aspx?nodeid=876&pid=4900). IPAQ-S asks about activities undertaken in leisure time, domestic and gardening (yard), work-related, and transport-related PA in the past seven days. The structured items in the IPAQ-S provide separate scores on walking, moderate-intensity and vigorous-intensity activity.

The intensity of a PA would be expressed in metabolic equivalents (METs). According to the official IPAQ scoring protocol, MET-min per week for an activity were calculated as MET values (vigorous 8.0, moderate 4.0, walking 3.3) x min of activity per day x days per week, and we summed the total PA MET-min/week for each participant^22^. Three levels of PA were used to classify the participants: low, moderate and high.

Since the amount of PA that WHO recommended for older adults equaled to 600 MET-min/week, we decided to group PA into two categories: 1. Low PA and 2. Moderate to high PA.

### Measurement of demographic factors, clinical factors and comorbidities

The questions listed in the questionnaire of the health checkup program include sex, age (birthday), current smoking status [Have you smoked in the last half year? (1) Never; (2) Seldom, social smoking; (3) ≤ 1 pack per day; and (4) > 1 pack per day. Answer 1 was classified as ‘No’; other answers were classified as ‘Yes’], alcohol drinking [Have you drank alcohol in the last half year? (1) Never; (2) Seldom, social drinking; and (3) Often. Answer 1 was classified as ‘No’; other answers were classified as ‘Yes’], education levels [We classified the items into 3 categories: (1) ≤ elementary school, (2) junior and senior high school, and (3) ≥ university], history of diseases (hypertension, diabetes mellitus, hyperlipidemia, cardiovascular disease, heart disease) and so on. If the participants were living in long-term care institutions, we got tabulations from institutions. Body height and body weight were measured and then automatically converted to BMI after recording the data into the system.

The participants’ blood samples have been collected during the health exam. We used the results of albumin, uric acid, fasting plasma glucose (FPG), total cholesterol, low-density lipoprotein cholesterol (LDL-C), high-density lipoprotein cholesterol (HDL-C), triglyceride (TG), and hemoglobin (Hb) as health indices and covariates.

### Statistical analyses

RStudio macOS Version 1.1.456 was used for statistical analysis. SAS 9.4 was used to double check the results. For descriptive statistics, we used χ^2^ test for categorical variables and t test or Mann–Whitney U test for continuous variables. Multiple logistic regression was used to analyze the relationship between PA and sarcopenia. Two-sided P < 0.05 was considered to be statistically significant. We set the level of power at 0.8 and used the software G^*^Power macOS version 3.1.9.6 to calculate the sample size before we recruited the participants.

Potential associated factors of sarcopenia were considered in the statistical analysis included age, BMI, education level, FPG, serum albumin, uric acid, total cholesterol, LDL-C, HDL-C, TG, Hb, alcohol drinking, smoking, institutionalization, and the history of diabetes mellitus, hypertension, dyslipidemia, cardiovascular disease, and heart disease. First, we did univariate analysis between each variable and sarcopenia (Table 2), if there was group-difference, we put those variables into the multiple logistic regression model. Odds ratios and corresponding 95% confidence intervals were calculated.

## Results

**Figure 1:**
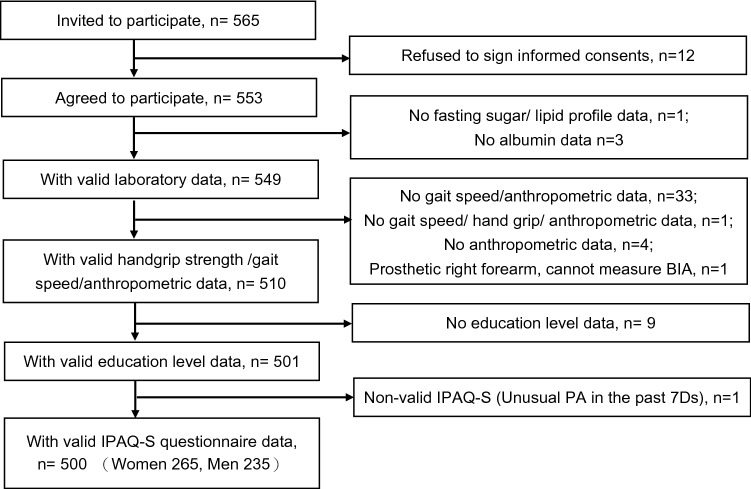
Study flowchart.

565 participants were recruited, 12 participants were excluded due to refused to participate and 53 were due to missing data, and 500 participants were used for the analysis (Fig. 1). The study participants had a mean age of 73.87 years old, with 47% men and 53% women. For both genders, the majority of participants’ PA levels were moderate, with women (142/265, 53.5%) and men (131/235, 55.7%). The mean of handgrip was 19.54 kg in women and 31.43 kg in men. The mean of usual gait speed was 0.99 m/s in women and 1.03 m/s in men. The mean of ASMI was 5.75 kg/m^2^ in women and 7.20 kg/m^2^ in men. We compared the demographic and clinical characteristics between low PA and moderate to high PA groups for all of the participants (Table 1).

**Table 1:** Basic demographic and clinical characteristics of the study participants.

	Low PA (n = 106)	Moderate to high PA^a^ (n = 394)
**Age, years**		
Mean (SD)	75.0 (6.79)	73.6 (6.62)
Median (IQR)	74.0 (11)	72.0 (10)
**Gender, n (%)**		
Women	73 (68.9%)	192 (48.7%)
Men	33 (31.1%)	202 (51.3%)
**Body mass index (BMI)**		
Mean (SD)	23.5 (3.31)	23.9 (3.22)
Median (IQR)	23.2 (4.2)	23.6 (3.775)
**Institutionalization**		
No	91 (85.8%)	379 (96.2%)
Yes	15 (14.2%)	15 (3.8%)
**Alcohol drinking**		
No	97 (91.5%)	321 (81.5%)
Yes	9 (8.5%)	73 (18.5%)
**Smoking**		
No	100 (94.3%)	374 (94.9%)
Yes	6 (5.7%)	20 (5.1%)
**Education level**		
1 ≤ elementary school	39 (36.8%)	88 (22.3%)
2 Junior/senior high school	23 (21.7%)	138 (35.0%)
3 ≥ University	44 (41.5%)	268 (68.0%)
**Past history**		
Hypertension	43 (40.1%)	138 (35%)
Diabetes mellitus	14 (13.2%)	56 (14.2%)
Hyperlipidemia	24 (22.6%)	78 (19.8%)
Cardiovascular disease	10 (9.4%)	36 (9.1%)
Heart disease	18 (17.0%)	35 (8.9%)
**Albumin, g/dL**		
Mean (SD)	4.39 (0.231)	4.40 (0.243)
Median (IQR)	4.40 (0.3)	4.40 (0.2)
**Uric acid, mg/dL**		
Mean (SD)	5.37 (1.30)	5.55 (1.40)
Median (IQR)	5.30 (1.975)	5.50 (2.1)
**Total cholesterol, mg/dL**		
Mean (SD)	179 (33.0)	181 (31.5)
Median (IQR)	176 (43)	179 (43)
**Fasting plasma glucose (FPG), mg/dL**		
Mean (SD)	103 (21.7)	103 (20.8)
Median (IQR)	98.0 (12.75)	99.0 (15)
**LDL-C, mg/dL**		
Mean (SD)	102 (28.9)	103 (27.8)
Median (IQR)	98.5 (36.5)	102 (36)
**HDL-C, mg/dL**		
Mean (SD)	55.3 (12.7)	55.9 (14.3)
Median (IQR)	54.5 (15)	53.0 (18.75)
**Triglyceride (TG), mg/dL**		
Mean (SD)	108.4 (49.6)	109.3 (50.6)
Median (IQR)	101 (54)	101 (60)
**Hemoglobin, g/dL**		
Mean (SD)	13.2 (1.21)	13.5 (1.35)
Median (IQR)	13.2 (1.38)	13.6 (1.6)

*PA* physical activity, *SD* standard deviation, *IQR* interquartile range, *LDL-C* low-density lipoprotein cholesterol, *HDL-C* high-density lipoprotein cholesterol.

^a^Physical activity level according to IPAQ category.

A total of 138 (27.6%) participants were classified as having sarcopenia (Table 2). Compared with the low PA group, the OR of moderate to high PA to sarcopenia was 0.46 (95% CI 0.27–0.79, p-value = 0.005) after adjusting for sex, institutionalization, age, BMI, albumin, hemoglobin, HDL-C levels, history of cardiovascular disease, education level and alcohol drinking (Table 3). There was only 30 institutionalized people, so we also did multiple logistic regression for those living outside the residential institutions (Table 4a,b).

**Table 2:** Comparisons of covariates in non-sarcopenia and sarcopenia participants according to the 2019 AWGS diagnostic criteria of sarcopenia.

	Non-Sarcopenia (N = 362)	Sarcopenia (N = 138)	P-value
**Physical activity**			< 0.001
Low	58 (54.7%)	48 (45.3%)	
Moderate to high	304 (77.2%)	90 (22.8%)	
**Gender**			
Women	178 (67.2%)	87 (32.8%)	0.007
Men	184 (78.3%)	51 (21.7%)	
**Institutionalization**			0.002
No	348 (67.8%)	122 (32.2%)	
Yes	14 (46.7%)	16 (53.3%)	
**Alcohol drinking**			0.014
No	293 (70.1%)	125 (29.9%)	
Yes	69 (84.1%)	13 (15.9%)	
**Smoking**			0.761
No	342 (67.1%)	132 (32.9%)	
Yes	20 (76.9%)	6 (23.1%)	
**Education level**			0.011
≤ Elementary school	79 (62.2%)	48 (37.8%)	
Junior/senior high school	124 (77.0%)	37 (23.0%)	
≥ University	159 (75.0%)	53 (25.0%)	
**Medical history—hypertension**			0.472
No	227 (71.2%)	92 (28.8%)	
Yes	135 (74.6%)	46 (25.4%)	
**Medical history—diabetes mellitus**			0.271
No	307 (71.4%)	123 (28.6%)	
Yes	55 (78.6%)	15 (21.4%)	
**Medical history—hyperlipidemia**			0.161
No	282 (70.9%)	116 (29.1%)	
Yes	80 (78.4%)	22 (21.6%)	
**Medical history—cardiovascular disease**			0.045
No	335 (73.8%)	119 (26.2%)	
Yes	27 (58.7%)	19 (41.3%)	
**Medical history—heart disease**			1.000
No	324 (72.5%)	123 (27.5%)	
Yes	38 (71.7%)	15 (28.3%)	
Age	72.7 (6.14)	76.9 (7.06)	< 0.001
Body mass index (BMI)	24.4 (3.24)	22.1 (2.54)	< 0.001
Albumin	4.41 (0.241)	4.34 (0.232)	0.003
Uric acid	5.58 (1.36)	5.33 (1.43)	0.074
Total cholesterol	180 (30.9)	182 (34.0)	0.413
Fasting plasma glucose (FPG)	104 (21.0)	102 (20.7)	0.392
Low-density lipoprotein cholesterol (LDL-C)	103 (27.3)	103 (29.9)	0.894
High-density lipoprotein cholesterol (HDL-C)	54.8 (13.4)	58.2 (15.2)	0.014
Triglyceride	110 (51.1)	105 (48.3)	0.257
Hemoglobin	13.6 (1.38)	13.1 (1.07)	< 0.001

**Table 3:** Adjusted model for associated factors of sarcopenia in the study population according to the 2019 AWGS diagnostic criteria of sarcopenia.

N = 500	Odds ratio (95% CI)	P-value
Moderate to high physical activity	0.46 (0.27, 0.79)	0.005
Sex (men)	0.45 (0.26, 0.80)	0.006
Age	1.11 (1.07, 1.15)	< 0.001
Body mass index (BMI)	0.74 (0.67, 0.81)	< 0.001
Institutionalization	2.58 (0.96, 6.99)	0.061
Albumin	0.44 (0.15, 1.24)	0.119
Hemoglobin	1.05 (0.85, 1.29)	0.648
High-density lipoprotein cholesterol (HDL-C)	1.00 (0.98,1.02)	0.978
Medical history of cardiovascular disease	1.93 (0.92, 4.03)	0.080
Education level	1.04 (0.76, 1.42)	0.827
Alcohol drinking	0.63 (0.30, 1.27)	0.214

**Table 4:** Adjusted model for associated factors of sarcopenia in the study population according to the 2019 AWGS diagnostic criteria of sarcopenia after excluding institutionalized people.

**N = 470**^**a,b**^	**Odds ratio (95% CI)**	**P-value**
**(a)**		
Moderate to high physical activity	0.45 (0.25, 0.79)	0.006
Sex (men)	0.44 (0.24, 0.78)	0.006
Age	1.11 (1.07, 1.15)	< 0.001
Body mass index (BMI)	0.73 (0.66, 0.81)	< 0.001
Albumin	0.42 (0.14, 1.24)	0.120
Hemoglobin	1.09 (0.87, 1.37)	0.462
High-density lipoprotein cholesterol (HDL-C)	1.00 (0.98, 1.02)	0.737
Medical history of cardiovascular disease	1.90 (0.85, 4.19)	0.112
Education level	1.08 (0.78, 1.51)	0.637
Alcohol drinking	0.63 (0.28, 1.30)	0.226
**(b)**		
Moderate to high physical activity	0.44 (0.25, 0.79)	0.006
Sex (men)	0.46 (0.26, 0.81)	0.008
Age	1.12 (1.08, 1.16)	< 0.001
Body mass index (BMI)	0.73 (0.66, 0.81)	< 0.001
Albumin	0.42 (0.14, 1.24)	0.137
Hemoglobin	1.10 (0.89, 1.39)	0.363
High-density lipoprotein cholesterol (HDL-C)	1.00 (0.98,1.02)	0.752
Alcohol drinking	0.63 (0.29, 1.29)	0.221

^a^Including all covariates as the whole 500 people model.

^b^Just including covariates those existed group-difference when we did univariate analysis (no education level and medical history of cardiovascular disease).

## Discussion

In this study, we observed that a significant protective effect of PA on sarcopenia was found among the older adults after adjusting for confounders. The prevalence of sarcopenia in this study population and our main findings were similar to most cross-sectional studies^5^. The prevalence was higher in women (32.8%) than men (21.7%). The relationship between PA, sex, aging, BMI and sarcopenia were consistent with most of previous studies^19^. Furthermore, Strong protective effect of PA and male were noticed, whereas higher BMI also showed a protective effect on sarcopenia.

Body muscle mass and strength are different in men and women (men greater than women) by nature. It is speculated that sex-related difference in regulation of muscle contraction may result in the more obvious frailty and impairment of muscle function in old women than in old men^23^. In another aspect, the outcomes in the exercise status survey by the Sports Administration of Ministry of Education in 2019 revealed that there was sex-difference in the intensity and frequency of exercise (men greater than women) in Taiwan^24^. Due to the reasons above, sex may affect both physical activity and sarcopenia and we regarded it as a confounder to explore the relationship between physical activity and sarcopenia. In our study, male is a strong protective factor of sarcopenia. Consequently, when we advocate the importance of sarcopenia, we can tell the women pay attention to do extra effort against sarcopenia.

We tried to search similar cross-sectional studies using structural questionnaires to evaluate PA and both of muscle mass and function to diagnose sarcopenia. Compare to previous Asian studies, one China study showed PA was not related to sarcopenia^25^. In one Korean study, vigorous and moderate PA were not associated with sarcopenia, but if PA displayed in quantiles, the third and fourth quantiles PA of the subjects showed protect effect on sarcopenia in Korean men^26^. On the other hand, in the western countries, one study in Peruvian Andes found that age, female sex, a low BMI, and little PA were associated factors of sarcopenia^27^. However, an Italian study showed nutritional intake, PA, and level of comorbidity were not associated with sarcopenia^28^. Nevertheless, a multi-continent study enrolled 18,363 people showed PA was a key factor for the prevention of sarcopenia^29^.

Although aging is the main cause of sarcopenia, it is an inevitable process. Since we can see the obvious protective effect of PA on sarcopenia, we should advocate regular physical activity to the public. Our study is unique in that we classified the PA according to WHO’s recommendation. Although previous researchers might explore the association between PA and sarcopenia via IPAQ, they classified the PA just according to the IPAQ protocol (into low, moderate and high), or by vigorous PA, moderate PA, and walking PA, or by quantiles^26^. Indeed, for additional benefits, older adults should increase their PA. However, in our daily clinical practice or health promotion activities, we found that when we tried to educate the older adults to do physical activity, some of them might refuse and mentioned that it was impossible for them to do ‘exercise’. Besides, some experts doubt that the PA level of at least 600 MET-min per week is enough. However, in our findings, the global recommendation of PA already showed obvious benefit to the older adults in the prevalence of sarcopenia. Through this study, we can apply our findings as an echo of WHO’s recommendation to educate the public that older adults can accumulate PA in their daily, family and community lives.

The moderate to high PA group was composed of participants with higher education level. Moreover, there was group-different when we did univariate analysis between education level and sarcopenia. However, the effect of education level on sarcopenia was not statistically significant (p-value = 0.827) in this study after we adjusted covariates. Although some studies showed the effect of education level on sarcopenia^28^, there is much more health-related knowledge spreading through social media and community care centers than before, so the health literacy may be elevated in the older adults regardless of their educational levels.

Sarcopenia prevalence are usually higher in long-term care institutions. Most of the residents may have several chronic diseases and comorbidities, who may have less PA compare to those living at homes. Hence, we expected to explore the association between institutionalization and sarcopenia based on the premise that confounders were adjusted. However, the effect of institutionalization on sarcopenia was not statistically significant (p-value = 0.061) in this study after we adjusted covariates. Although living in long-term care institutions similarly, those who can attend the health checkup program may be healthier compared to those who cannot or refuse to do so. Suppose we collect more institutionalized people, the association between institutionalization and sarcopenia may be clearer. Furthermore, we tried to exclude institutionalized people (N = 30) to analyze those individuals living in the community only (N = 470). The results were similar to which from the 500 participants together.

Metabolites, such as reactive oxygen species, reactive nitrogen species, and aldehydes are components of the cigarettes smoke, enter the bloodstream and arrive at the skeletal muscles of smokers and accelerate muscle wasting^30^. Based on the theory above, we assumed there should be association between smoking and sarcopenia. However, only 26 (25 men and 1 woman) participants were smokers and there was no statistically significance (p-value = 0.761) when we did univariate analysis, so we did not analyze the relationship. In the traditional Chinese society, it brought about a negative concept that a woman was a smoker. Consequently, some people might pretend that they were not smokers, which was one kind of social desirability bias. Moreover, the Taiwanese older generation did not have smoking habit originally, which reflected on that smoking rate was low (0.7% in women) in adults above 65 years old according to the 2018 Adult Smoking Behavior Surveillance System. It was reasonably that we only recruited one smoking woman.

With increased lean body mass loss, associated mortality increased, which even could up to 100% when one person has lost 40% lean body mass^3^. One previous study showed increased sarcopenia prevalence with decreased BMI^31^. The association between BMI and mortality has been revealed to have a U- or J-shaped configuration, with better health-related outcomes and longevity observed for older adults in the overweight category of the BMI classification^32^. In the past, the traditional concept has been that being thin leads to longevity. Nowadays, more and more geriatricians and dietitians are saying that extremely low BMI is related to higher mortality. Our study proved that higher BMI was a protective factor of sarcopenia. Hence, we can do some education to the underweight older adults, encouraging them to keep a suitable weight for better health.

One of the strengths of our study was including demographic and clinical factors. Additionally, our study consisted of international physical activity and sarcopenia assessment tools, which can be compared with other countries. To our best knowledge, this is the first Taiwanese data using the latest AWGS diagnostic criteria to analyze the participants. We can use the result to appeal to the health care professionals to pay attention to the increasing sarcopenia population.

Our study had several limitations. First of all, it was a cross-sectional study which only revealed the association between PA and sarcopenia, but not illustrating the cause-effect relationship. In addition, the participants were recruited during the health exam and there might be a selection or sampling bias due to healthy user effect. Since this was a hospital-based study, rather than a community-based study, we just did convenience sampling. Hence, we could not collect more institutionalized participants or smokers. Therefore, we could not explore the relationship between institutionalization and sarcopenia, and smoking and sarcopenia. The participation bias might exist and thus the results cannot be extrapolated to the general population.

Population aging is a trend. We should reminder the public that there were about 1 out of  3 old women and 1 out of  5 old men in our community had sarcopenia. The ratios are considerable and unimaginable to the public. Aging and sex are unchangeable, but physical activity can be done. The higher amount of physical activity was associated with about half odds ratio of sarcopenia. We can apply the results in this study to tell the older adults that physical activity is a key toward sarcopenia as long as they will try. Health professionals can design special courses and encourage women do more physical activity. Especially for the fragile older adults, we can set the least goal and inspire them to accumulate physical activity in daily life. Intervention studies such as vigorous-intensity or frequent physical activity for who already meet the diagnostic criteria of sarcopenia can be carried out. In the future, researchers can also try to analyze the effect of long-term physical activity habit, such as since youth, on sarcopenia.

## Conclusions

A significant protective effect of physical activity on sarcopenia was found among the older adults after adjusting for confounders. Higher BMI and male sex also showed protective effect on sarcopenia, while aging was a risk factor of sarcopenia. Further cohort studies and even RCTs may be needed to confirm our findings.

## Data Availability

The datasets generated and/or analyzed during the current study are not publicly available due to legal restrictions imposed by the government of Taiwan in relation to the “Personal Information Protection Act”.
